# Effects of Caloric Restriction and Exercise Training on Skeletal Muscle Histochemistry in Aging Fischer 344 Rats

**DOI:** 10.1100/tsw.2006.183

**Published:** 2006-10-13

**Authors:** David T. Lowenthal, Zebulon V. Kendrick, Joseph W. Starnes, Eli Carmeli

**Affiliations:** ^4^ Department of Physical Therapy and Skeletal Muscle Laboratory, Sackler Faculty of Medicine, Stanley Steyer School of Health Professions, Tel Aviv University, Ramat Aviv, Israel; ^1^ (GRECC) Geriatric Research, Education and Clinical Center, Department of Veterans Affairs Medical Center, 1601 SW Archer Road, Gainesville, FL, USA; ^2^ Research Laboratory, College of Health Professions, Temple University, Philadelphia, PA, USA; ^3^ Department of Kinesiology and Health Education, Cardiac Metabolism Laboratory, University of Texas, Austin, TX, USA

**Keywords:** skeletal muscle, caloric restriction, running, aging

## Abstract

The purpose of this study was to determine the effects of calorie restriction and exercise on hindlimb histochemistry and fiber type in Fischer 344 rats as they advanced from adulthood through senescence. At 10 months of age, animals were divided into sedentary fed *ad libitum*, exercise (18 m/min, 8% grade, 20 min/day, 5 days/week) fed *ad libitum*, and calorie restricted by alternate days of feeding. Succinic dehydrogenase, myosin adenosine triphosphatase (mATPase at pH 9.4), nicotine adenonine dinucleotide reductase, and Periodic Acid Shiff histochemical stains were performed on plantaris and soleus muscles. The results indicated that aging resulted in a progressive decline in plantaris Type I muscle fiber in sedentary animals, while exercise resulted in maintenance of these fibers. The percent of plantaris Type II fibers increased between 10 and 24 months of age. Exercise also resulted in a small, but significant, increase in the percentage of plantaris Type IIa fibers at 24 months of age. The soleus fiber distribution for Type I fibers was unaffected by increasing age in all groups of animals. The implications of these results suggest the implementation of exercise as a lifestyle modification as early as possible.

## INTRODUCTION

Recently, there have been trends toward increased physical activity in older individuals; however, the majority of elderly people do not exercise and indeed are often discouraged from participating in regular activity. Physical capacity has been observed to decline with increasing age in humans[1,2,3,4] as well as in animals[5,6]. Yet improvement in aerobic capacity has been demonstrated once an exercise regimen is initiated and maintained from at least 9 weeks[7] to a 4- to 6-month period in humans to achieve health benefits[8,9,10] and to improve the regional blood flow following 2–3 months of exercise training in late-middle-aged rats[11,12]. Aging confers resiliency and is capable of responding to an aerobic stimulus. Nonetheless, it remains to be determined whether the loss of physical capacity is related to age or is related to the reduction of physical activity common in older individuals. The decline in function is due to both deconditioning (due to less physical activity) and to aging effects.

Long-term studies of aging in animals undergoing caloric restriction have been underway for over a decade. The diverse effects of caloric restriction have been demonstrated many hundreds of times in laboratory rodents and other short-lived species. In animals, caloric restriction has multiple effects on a variety of organ systems. Caloric restriction and physical activity are the only intervention that reproducibly extends mean and maximal life span in short-lived mammalian species[13]. Exercise training and caloric restriction produce similar changes in body composition[14]. Studies of various markers related to age-related diseases suggest that caloric restriction as well as physical activity will prevent or delay the onset of cardiovascular disease, diabetes, and perhaps cancer.

The usefulness of the Fischer 344 rat is an excellent model to study many questions in gerontology and has been extensively reviewed[15]. The use of Fischer 344 rats has been criticized because of their high incidence of particular age-related diseases[16]. However, research in exercise physiology using the F344 rats can be extrapolated to human performance[17,18]. Age-related reductions in oxidative energy production were observed in the gastrocnemius muscle of sedentary animals lessened with the exercise intervention. Rumsey et al.[18] concluded that the age-associated decrement in energy production of the gastrocnemius muscle was more likely due to age-induced decrements in locomotion than due to decreases in energy levels. Biochemical analyses and enzyme histochemical techniques have provided evidence for skeletal muscle plasticity, biochemical changes, and fiber type transformation as influenced by exercise in aging animals and humans[19,20].

The purpose of this study was to do a long-term cross-sectional assessment on skeletal muscle enzymes, qualitatively, by histochemistry and skeletal muscle fiber type in sedentary, exercise, and calorie-restricted states at varying ages ranging from 10–30 months. Lastly, the extended purpose of this study was to do a long-term analysis on the effects of continuous aerobic exercise on skeletal muscle by enzyme histochemistry.

## METHODS

Nine month-old male Fischer 344 rats (retired breeders and specific pathogen-free) were obtained from the Charles River Breeding Laboratories (Kingston, NY) and housed thereafter as sole occupants in a room maintained at 21 ± 1$^{o}$C on a 12-h light/dark cycle. Animals were paired-caged and rested on hardwood shavings in 25- × 45- × 22-cm plastic cages with covered filtered bonnets.

Following a 30-day acclimatization period, animals were randomly divided into three groups (n = six animals started in each group, at ages of 10, 18, 24, 30 months. Table 1 shows the total number left during the course of the study): sedentary, fed *ad libitum* fed (SF); exercise, fed *ad libitum* (EF); and sedentary, calorie restricted (SCR) (i.e., the animals every other day such that their caloric intake was 60% of that of *ad libitum* fed). Exercised animals were forced to run on a motor-powered treadmill (18 m/min, 8% grade, 20 min/day, 5 days/week from 10–25 months of age and at 12 m/min between 25 and 30 months of age, because some animals were having difficulty maintaining the 18 m/min speed for the 20 min).

Animals were maintained on their protocols until sacrificed at 18, 24, and 30 months of age. Animals were fasted overnight prior to sacrifice, which occurred between 11:30 and 13:30 h.

Animals were anesthetized by intraperitoneal injection of Na-pentobarbital (40 mg/kg body weight). The gastrocnemius-plantaris-soleus groups of muscle from both hindlimbs were exposed and removed by severing the proximal and distal attachments. The muscles were rinsed in ice-cold homogenization medium, isolated, and dissected of fat and tendons on an ice-cold glass petri dish. After blotting of the right hindlimb plantaris and soleus, wet weights were recorded and the muscles were placed in cooled liquid isopentane, then transferred to liquid nitrogen until histochemical analyses were done.

**TABLE 1**
**Effect of Age, Exercise, and Calorie Restriction on Body Mass and the Masses of the Plantaris and Soleus Muscles**

| | Age, Months | | | |
|---|---|---|---|---|
| **Variable/Condition** | **10** | **18** | **24** | **30** |
| Body Weight (g) | | | | |
| Sedentary | 374 ± 9 | 442 ± 11 | 413 ± 18 | *364 ± 19 |
| No. of rats | (6) | (5) | (5) | (5) |
| Exercise | 375 ± 5 | 361 ± 6 | 332 ±15 | |
| | (6) | (5) | (5) | |
| Calorie Restricted | 342 ± 9 | 353 ± 7 | *308 ± 17 | |
| | (6) | (5) | (4) | |
| Plantaris Muscle (mg) | | | | |
| Sedentary | 348 ± 11 | 384 ± 13 | 373 ± 5 | *297 ± 17 |
| No. of muscle tissues | (8) | (8) | (8) | (8) |
| Exercise | 341 ± 7 | 329 ± 7 | 309 ± 17 | |
| | (8) | (8) | (8) | |
| Calorie Restricted | 308 ± 8 | 303 ± 8 | 281 ± 9 | |
| | (8) | (8) | (8) | |
| Soleus Muscle (mg) | | | | |
| Sedentary | 163 ± 3 | 153 ± 6 | 141 ± 6 | *131 ± 9 |
| | (8) | (8) | (8) | (8) |
| Exercise | 41 ± 5 | 129 ± 4 | *109 ± 3 | |
| | (8) | (8) | (8) | |
| Calorie Restricted | 134 ± 4 | 126 ± 4 | 119 ± 4 | |
| | (8) | (8) | (8) | |

Values are means ± SEM. In the parenthesis are the “observations” that are the numbers of animals in each group. *$p < 0.05$.

Sequential slices of plantaris and soleus muscles were cut on a freezing microtome (2–4ºC) at a thickness of 8–10 µm and prepared for histochemical fiber type analyses[21]. For mitochondrial content, we used succinic dehydrogenase (SDH)–stained sections[22]. The other enzyme stains were alkaline (pH 9.4) and reverse myosin ATPase (pH 4.3)[23] and nicotine adenonine dinucleotide reductase (NADPH)[24]. These stains are appropriate for identifying Type I, Type IIa, and Type IIb muscle fibers. Periodic Acid Schiff (PAS)[21] for qualitative glycogen content was also undertaken to identify age-associated differences in muscle glycogen concentration in these muscles. Both Type I and Type II muscle fibers contain glycogen, with Type II muscle fibers generally having greater amounts of glycogen.

Approximately 130–150 randomized muscle fibers were counted from a Polaroid photograph taken of the muscle section–prepared slide on a standard multistage Zeiss microscope (MIDSCI, St. Louis, MO) at varying magnifications. The photograph covers the entire cross-section of each muscle and the observer was blinded to the identity of the samples. This means of analysis allowed for ease of visibility because the photograph can be more magnified than microscopic visualization of the tissue under high-power lens. The determination of the distribution of Type I, Type IIa, and Type IIb muscle fibers was verified by independent observers skilled in neuromuscular anatomy.

### Statistical Analyses

A 1 × 4 analysis of variance (ANOVA) was performed to determine differences among ages for each of the three groups of animals. To discriminate among SF and EF animals, a 2 × 3 ANOVA was performed. When the F ratios indicated significant differences, these differences were located with the Newman-Keuls post-hoc test. A minimal level of confidence for all statistical analyses was established at $p < 0.05$. The Student *t*-test was used where appropriate.

## RESULTS

### Morphometric Changes with Age

Body weight of each group of animals declined significantly in response to senescence with the greatest decline between 24 and 30 months of age (see Table 1). It is noteworthy that the decline in body weight occurred at the time when food consumption was stable until at least 26 months of age (data not reported). The significantly lower body weight of EF and SCR animals was more than SF animals at 18, 24, and 30 months of age, which demonstrated the efficacy of the increased caloric expenditure of exercise and the caloric restriction, respectively.

Greater differences in loss of body weight and plantaris muscle weight between 18 and 30 months of age were observed for the SF (18 and 23%) than for the EF (11.5 and 9.4%) and (9.9 and 8.8) body and plantaris muscle weights, respectively. No significant reductions for plantaris muscle weight to body weight ratio was observed for SF animals between 10 and 30 months and 24 and 30 months of age (data not reported).

Progressive decreases in soleus muscle weight occurred between 18 and 30 months of age for SF animals whereas EF animal soleus weight remained relatively constant until 24 months of age. Soleus muscle weight to body weight ratio was significantly lower in SF animals than EF animals between 18 and 30 months of age.

### Enzyme Histochemistry of the Plantaris Muscle

The plantaris is composed of mixed fiber composition. The composite distribution of Type IIa (fast twitch oxidative, glycolytic), IIb (fast twitch glycolytic), and Type I (slow twitch oxidative) plantaris muscle fibers of sedentary fed and exercise fed animals at 10, 18, 24, and 30 months of age are found in Table 2.

Tables 1 and 3 depict composite age-associated and exercise effects on Type I, IIa, and IIb muscle fibers as determined by the stain SDH. Table 3 depicts 30-month-old muscle and calorie-restricted condition as determined by the stain PAS. Table 3 depicts 30-month-old muscle and exercise condition as determined by the stain NADH. The percent of Type IIb fibers increased in sedentary fed animals between 10 and 24 months of age, which was maintained between 24 and 30 months of age. The percent of Type I fibers decreased between 10 and 24 months of age with a further reduction in the percent of Type I fibers between 24 and 30 months of age.

Figs. 1a, 1b, 2, 3, 3a depict composite age-associated and exercise effects on type I, IIa, and IIb muscle fibers as determined by the stain for SDH. Fig. 4a and b depict 30-month-old muscle and exercise condition as determined by the stain for PAS, respectively. The percent of Type IIb fibers increased in sedentary fed animals between 10 and 24 months of age, which was maintained between 24 and 30 months of age. The percent of Type I fibers decreased between 10 and 24 months of age with a further reduction in the percent of Type I fibers between 24 and 30 months of age.

**TABLE 2**
**Effect of Exercise on Plantaris Muscle Fiber Distribution of Age-Matched Animals**

| Condition | Age | Percent Fiber Distribution | | |
|---|---|---|---|---|
| | | Type IIa | Type IIb | Type I |
| Sedentary | 10 | 27.4 ± 0.9 | 44.4 ± 1.4 | 27.8 ± 1.5 |
| Sedentary | 18 | 26.0 ± 1.6 | 50.1 ± 2.3 | 23.6 ± 2.1 |
| Exercise | 18 | 30.1 ± 2.0 | 41.2 ± 1.9* | 29.9 ± 1.4* |
| Sedentary | 24 | 27.1 ± 1.2 | 52.5 ± 1.4 | 20.4 ± 0.8 |
| Exercise | 24 | 30.8 ± 0.9* | 39.9 ± 0.8* | 29.1 ± 0.7* |
| Sedentary | 30 | 36.1 ± 1.3 | 53.8 ± 1.9 | 9.8 ± 1.2 |
| Exercise | 30 | 34.5 ± 1.6 | 41.0 ± 1.8* | 24.4 ± 1.1* |

Age in months. Values are means ± SEM. *Represents a significant difference ($p < 0.05$) from age-matched sedentary animals.

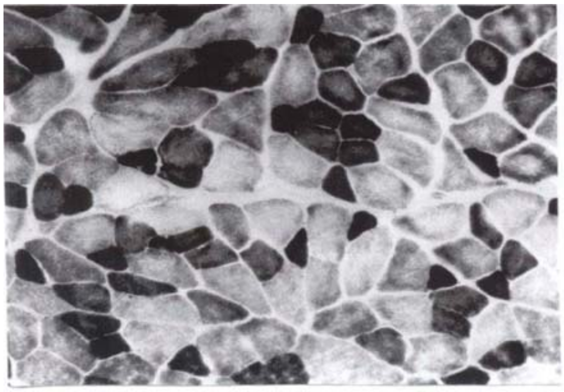

**Figure 1a.** SDH stain, plantaris muscle, 10-month old sedentary.

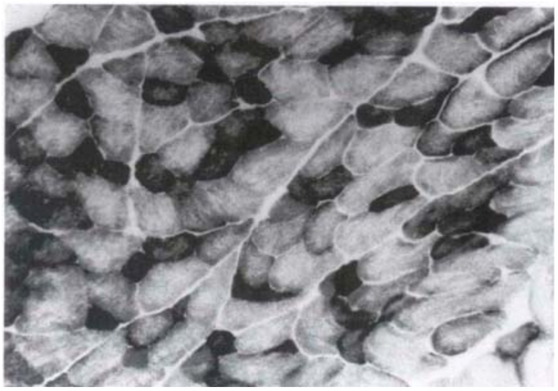

**Figure 1b**. SDH stain, plantaris muscle, 18-month old sedentary.

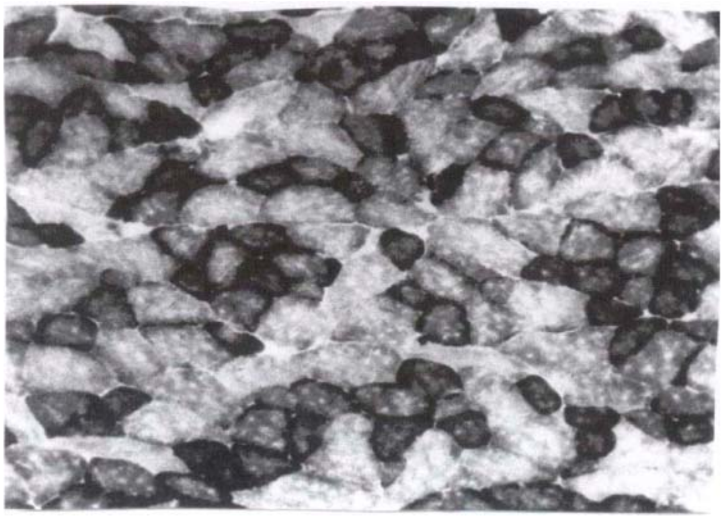

**Figure 2.** SDH stain, plantaris muscle, 24-month old sedentary.

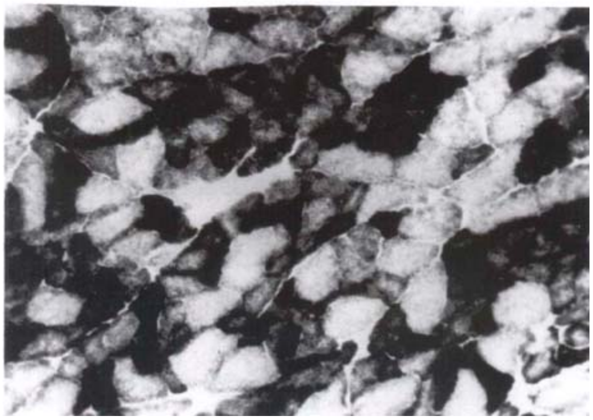

**Figure 3a.** SDH stain, plantaris muscle, 30-month old sedentary.

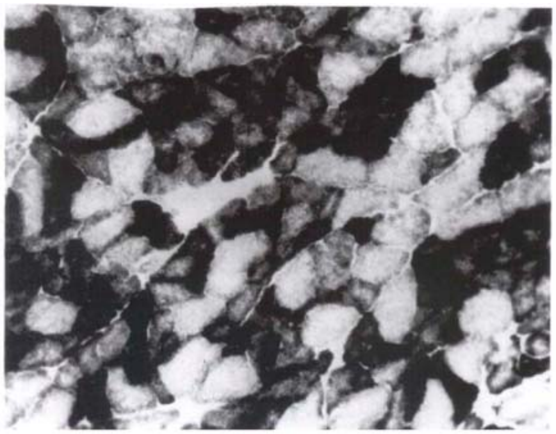

**Figure 3b.** SDH stain, plantaris muscle, 30-month old exercised.

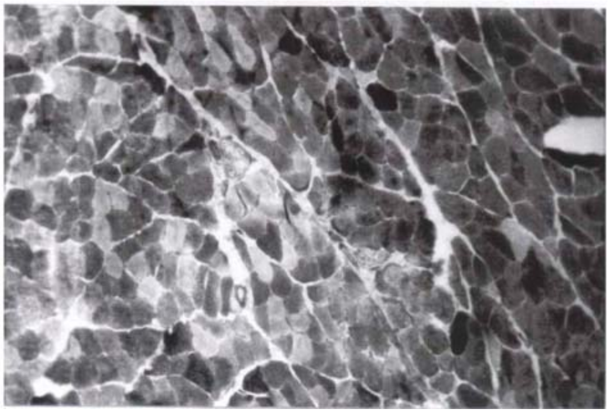

**Figure 4a.** PAS stain, plantaris muscle, 30-month old underfed

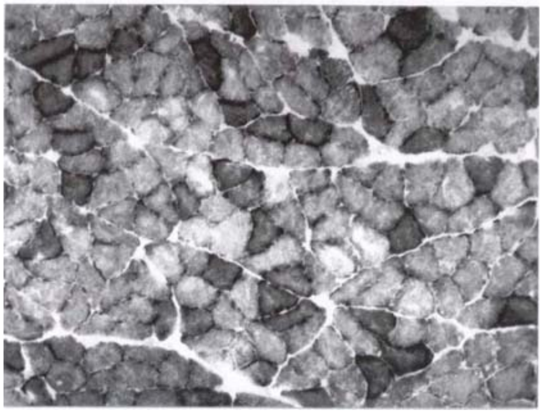

**Figure 4b**. NADH stain, plantaris muscle, 30-month old exercised.

The exercise intervention resulted in a small, but significant, decrease in the percent of Type IIb fibers between 10 and 24 months of age, and the percent of the Type IIa fibers increased between 10 and 24 months of age. The percent of Type I fibers did not significantly change between 10 and 30 months of age in the exercise animals.

Significant differences in the percent of fiber distribution between age-matched sedentary fed and exercise fed animals were observed for Type IIa fibers at 24 months of age, Table 2. Significant differences in the percent fiber distribution between sedentary fed and exercise fed animals were observed for Type IIb and Type I fibers at 18, 24, and 30 months of age.

## Enzyme Histochemistry of the Soleus Muscle

The soleus muscle contains a preponderance of type I fibers, and the remainder of the fibers are type II. Tables 1–3 depict the age-associated changes and those changes attributed to underfed (Tables 1–3, NADH stain), and changes attributed to exercise (Table 1–3, SDH and ATPase stains, respectively). The tables as well as Table 3 demonstrated that the soleus muscle fiber distribution was neither significantly influenced by advancing age or by exercise or calorie restriction.

Figs 5a (NADH stain), 5b (SDH stain), and 6 (ATPase stain) depict the age-associated changes and those changes attributed to underfed, and changes attributed to exercise. The figures as well as Table 3 demonstrate that the soleus muscle fiber distribution was neither significantly influenced by advancing age nor by either exercise or calorie restriction. The PAS stain (not presented) lacked specificity in the soleus muscle and no firm conclusions could be derived.

**Table 3**

**Percent Fiber Distribution of Age-Matched Soleus Muscle**

| | | Percent Fiber Distribution | | | |
|---|---|---|---|---|---|
| **Condition** | **Fiber Type** | **10** | **18** | **24** | **30** |
| Sedentary | I | 82.0 ± 6.8 | 84.8 ± 1.5 | 82.0 ± 2.6 | 77.3 ± 0.3 |
| | II | 18.0 ± 4.4 | 15.2 ± 1.5 | 18.0 ± 2.7 | 23.7 ± 2.9 |
| Exercise | I | | 85.3 ± 5.7 | 86.0 ± 1.9 | 84.4 ± 2.4 |
| | II | | 14.9 ± 5.9 | 14.0 ± 1.9 | 15.6 ± 2.4 |
| Calorie Restricted | I | | 87.5 ± 3.5 | 88.1 ± 2.8 | *79.8 ± 2.8 |
| | II | | 12.5 ± 3.7 | 11.9 ± 2.8 | *20.2 ± 2.3 |

Values are means ± SEM. *$p < 0.05$

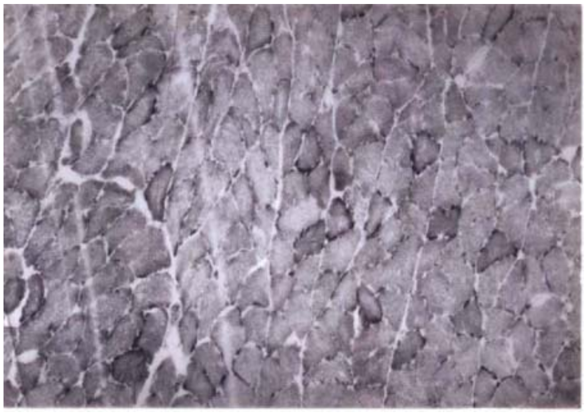

**Figure 5a.** NADH stain, soleus muscle, 18-month old underfed.

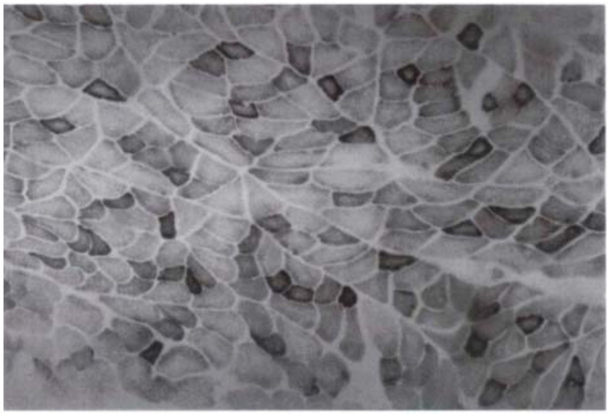

**Figure 5b**. SDH stain, soleus muscle, 10-month old, sedentary.

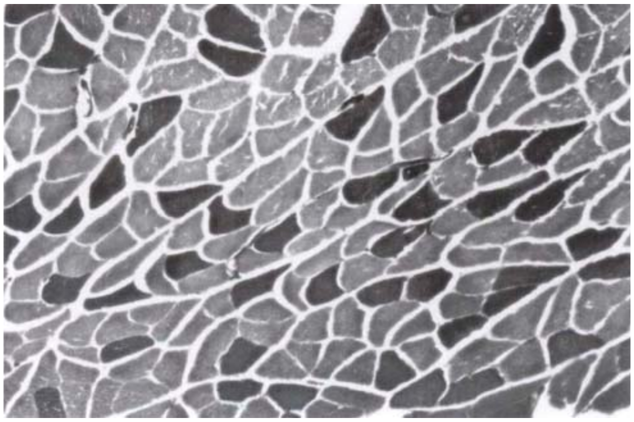

**Figure 6.** ATPase statin, pH 9.4, Soleus muscle, 10-month old sedentary.

## DISCUSSION

The results of this study showed that age-associated changes in body mass of sedentary fed animals parallel the findings of other investigations using the Fischer 344 rat[22]. The growth of the animals was nearly complete by 10 months of age. The implementation of both the exercise and the calorie-restricted lifestyle modifications resulted in the expected decline in body weight. From 10–24 months of age, the body weights of all three groups remained stable. Significant decline in body mass occurred after 24 months of age in all three groups of animals. Declines in the masses of the plantaris and soleus muscles followed similar patterns as body weight, suggesting that muscle atrophy was a contributing factor to loss of body mass. McCarter et al.[25] and others[26] have suggested that the sensitivity of skeletal muscle to atrophic changes may be causally linked to a more limited locomotion that occurs with senescence[27]. Voluntary movements are known to become fewer in the caged laboratory animal as it grows older[28]. Short-term limb immobilization, an extreme form of disuse, also results in muscle atrophy and a decreased capacity to oxidize both carbohydrate and lipid substrate in the affected limb[29]. The age-associated atrophy of the plantaris and soleus muscles in the study confirms the work of Holloszy et al. and others[30,31,32] of age-related decreases in muscle weight due to a decrease in number and/or diameter of muscle fibers.

Dietary energy restriction is the only proven method for extending lifespan and slowing aging in mammals, while maintaining health and vitality. In our experimental animals, we fed 40% less than controls, yet their diets were supplemented with micronutrients to achieve undernutrition without malnutrition. Those who were calorie restricted had less fat and lean mass than controls.

Aging results in a differential gene expression pattern specific to skeletal muscle tissue and most alterations can be completely or partially prevented by physical activity and by calorie restriction. Transcriptional patterns of tissues from calorie-restricted animals suggested that calorie restriction delayed the aging process by reducing endogenous damage and by inducing metabolic shifts associated with specific transcriptional profiles. Individual human and rat muscles have their own characteristic changes during senescence[20,33]. Type I slow twitch oxidative muscles show a reduction in number, whereas Type II demonstrate a reduction in volume and number. These changes may at times be random involving only focal groups of fibers[34].

The plantaris muscle is comprised of Type I, Type IIa, and IIb fibers, whereas the soleus muscle is principally comprised of Type I muscle fibers and was used as a control muscle whose fiber type has been shown to undergo no significant changes with age[31]. Type I muscle fibers are highly oxidative, slow twitch fibers[35,36], whereas Type IIa muscle fibers are fast oxidative, glycolytic fibers and Type IIb muscle fibers are fast glycolytic fibers[37,38]. Moreover, the plantaris and soleus muscles differ by more than simply their fiber type composition. They are also recruited under different circumstances, with the soleus being used during postural maintenance, whereas the plantaris is more important during locomotor behavior. Thus, changes in soleus muscle in sedentary animals are not very likely, yet changes in plantaris with aging could reveal the effects of both reduced recruitment and aging process.

In the present study, aging resulted in a progressive decline in the Type I slow oxidative muscle fibers of the plantaris muscle. The percentage of Type IIb muscle fibers increased between 10 and 24 months of age. The intervention of exercise resulted in a small, but significant, increase in the percentage of plantaris Type IIa fast twitch oxidative glycolytic muscle fibers at 24 months of age. The percentage plantaris IIb, FG, fibers decreased significantly as a result of the implementation of the exercise protocol. The exercise program resulted in a significant maintenance of the Type I fibers with the percent of Type I fibers remaining relatively stable between 10 and 30 months of age. The exercise-induced maintenance of Type I muscle fibers was consistent with the biochemical data reported by Rumsey et al.[18], who found the oxidative capacity of gastrocnemius muscle maintained between 10 and 24 months of age in exercise trained animals by Hammeren et al.[39].

The soleus muscle contains a preponderance of Type I muscle fibers. The soleus muscle fiber distribution for Type I fibers was not affected by age in all conditions. It is likely that the low intensity of our exercise protocol may be insufficient for any exercise induced change in the soleus muscle[31].

Chronic exercise has been well documented to improve the oxidative capacity of rat skeletal muscle[40]. Hammeren and colleagues[39] have shown that endurance training in old Fischer 344 rats increases both SDH and glutathione peroxidase (GPX) concentrations in soleus and red gastrocnemius, and a shift in myosin heavy chain (MHC) isoforms from a faster to a slower MHC in plantaris muscle[41]. These biochemical data support the observations that the maintenance of Type I muscle fibers is related to exercise in aging animals. The exercise protocol utilized in this study was sufficient to improve the oxidative capacity of the gastrocnemius muscle about 30% above that of the sedentary control animals. As previously observed by Holloszy[32], a reduction in food intake in exercising rats had a significant impact not seen in sedentary rats.

Correlating the histochemical data presented in this study with the biochemical data presented by Rumsey et al.[18] can be justified. Martin et al.[42] demonstrated correlation coefficients of 0.99 between SDH and alpha-glycerol phosphate dehydrogenase histochemical and biochemical activity determination. These investigators determined that biochemical units could be applied for these two particular histochemical stains. Although Rumsey et al.[18] did not determine SDH activity and alpha-glycerol phosphate, they did demonstrate that the exercise protocol utilized in this study resulted in the 30% increase in oxidative capacity of the mixed gastrocnemius muscle. Because the turnover number of cytochrome oxidase was similar in sedentary fed and exercise fed animals, Rumsey et al.[18] attributed the enhanced respiratory rates to increased mitochondrial content in the exercising muscle. Because oxidative fibers of muscle contain the greatest concentration of mitochondria and respiratory capacity[35,43], exercise-induced increase in and maintenance of Type IIa muscle fibers would be expected in the plantaris and soleus muscles.

## CONCLUSIONS

In the present study, exercise maintained the Type I muscle fibers of the plantaris muscle, when compared to the age-matched sedentary fed animals. Although Type I fiber distribution was greater in 30-month-old exercise fed than sedentary fed animals, the exercise paradigm could not prevent some of the age-associated loss of plantaris muscle. Rumsey et al.[38] observed a similar pattern with the oxidative capacity of the gastrocnemius muscle. It is clear that the early implementation of exercise as a lifestyle modification should result in better maintenance of muscle fiber types responsible for oxidative metabolism.

## ACKNOWLEDGMENTS

The author wishes to thank Scott Powers, PhD from Gainesville, Florida, for his thoughtful comments in the finalization of this manuscript and to Jenika Christmas for her secretarial assistance.
